# Pleural effusion as a mortality predictor for acute pulmonary embolism

**DOI:** 10.1016/j.rpth.2026.106857

**Published:** 2026-07-14

**Authors:** David Jiménez, Roger D. Yusen, Ignacio Jara, Francisco Galeano-Valle, Pablo Demelo-Rodríguez, Pedro Ruiz-Artacho, Giovanni Barillari, Olga Gavín-Sebastián, Behnood Bikdeli, Manuel Monreal, RIETE Investigators, A. Abad-Fernández, M.D. Adarraga, J. Aibar, A. Alda, J. Alfonso, J. Álvarez-Albarrán, C. Amado, M. Angelina-García, J.I. Arcelus, A. Ballaz, R. Barba, C. Barbagelata, M. Barrón, B. Barrón-Andrés, J. Bascuñana-Girón, F. Beddar-Chaib, E.I. Bencosme de Méndez, Á. Blanco-Molina, N. Chamorro, L. Chasco, G. Claver, J. Creu-Paris, J. Criado, C. De Juana-Izquierdo, F. Del Molino, J. Del Toro, P. Demelo-Rodríguez, M.C. Díaz-Pedroche, J.A. Díaz-Peromingo, Á. Dubois-Silva, D. Durán, J.C. Escribano, C. Falgá, L.A. Fernández-Bermejo, C. Fernández-Martínez de Septién, J.L. Fernández-Reyes, J. Ferreiro-Celeiro, C. Gabara, F. Galeano-Valle, A. García-Ortega, O. Gavín-Sebastián, A. Gil-Díaz, C. Gómez-Cuervo, J. Gorostidi-Pérez, L. Guirado, J. Gutiérrez-Guisado, L. Hernández-Blasco, J. Hernández-Borge, D. Jiménez, M.D. Joya, P. Llamas, H. López-Brull, L. López-Jiménez, P. López-Miguel, J.J. López-Núñez, A. López-Ruiz, J.B. López-Sáez, A. Lorenzo, O. Madridano, A. Maestre, P.J. Marchena, C. Menéndez-Sánchez, M.I. Mercado, J. Moisés-Lafuente, M. Monreal, L. Monzón-Escribano, A. Moreno-Fernández, M.S. Navas, J.A. Nieto, M.J. Núñez-Fernández, M. Olid, L. Ordieres-Ortega, M. Ortiz, J. Osorio, C. Osoro-Suárez, S. Otálora, J. Pagán, P. Parra-Caballero, J.M. Pedrajas, M. Pérez-Pinar, C. Pérez-Ductor, M.L. Peris, M.L. Pesce, J.A. Porras, L.M. Prieto-Gañán, G. Puche, D. Revilla, A. Rivas, F. Rivera-Cívico, A. Rivera-Gallego, M. Roca-Toledo, A. Rodríguez-Cobo, P. Ruiz-Artacho, N. Ruiz-Giménez, M. Sánchez-Camacho, T. Sancho, G. Sierra-Palomares, P. Sigüenza, L. Sindín-Martín, A. Solé, S. Suárez-Fernández, R. Tascón-Rodríguez, L. Terés-Pueyo, R. Tirado, M.R. Toda, C. Tolosa, P. Torregrosa-Ruíz, J. Trujillo-Santos, L. Tudela, R. Valle, J.F. Varona, E. Vega-Romero, G. Vidal, P. Villares, J.S. You, C. Ay, S. Nopp, I. Pabinger, M. Erard, Q. Van Thillo, P. Verhamme, A.T. Rocha, H.H.B. Yoo, S.E. Carbonell, J.E. Gómez-Mesa, A.C. Montenegro, J. Roa, P. Grenar, J. Hirmerova, R. Malý, T. Válková, S. Accassat, L. Bertoletti, A. Bura-Riviere, J. Catella, R. Chopard, F. Couturaud, B. Crichi, O. Espitia, R. Le Mao, B. Leclercq, I. Mahé, G. Millet, F. Moustafa, L. Plaisance, G. Poenou, G. Sarlon-Bartoli, E. Versini, Y. Jenab, A. Khodayari, P. Sadeghipour, S. Yadangi, S. Schellong, B. Brenner, I. Tzoran, G. Barillari, M. Basaglia, F. Bilora, D. Bissacco, C. Bortoluzzi, M. Bortoluzzi, B. Brandolin, G. Buso, R. Casana, M.M. Ciammaichella, P. Di Micco, A. Guida, E. Imbalzano, D. Lambertenghi-Deliliers, A. Licci, C. Marcon, D. Mastroiacovo, U. Mazzarelli, N. Mumoli, R. Pesavento, V. Pizzuti, A. Poz, P. Prandoni, P. Scarinzi, C. Siniscalchi, A. Visonà, N. Vo Hong, B. Zalunardo, H. Cesnieks, D. Kigitovica, A. Skride, C. Flores-Ramírez, M.Á. Mendoza Romo-Ramírez, D. Tarín-Recéndez, S. Fonseca, M. Martins, C. Pereira-Fontes, B. Soeiro, M. Bosevski, M. Zdraveska, S. Barco, L. Mazzolai, D.J. Angiolillo, B. Bikdeli, J.A. Caprini, A. Khalil, L. Ortega-Paz, G.M. Parmar, A.J. Tafur, M.H. Bui, S.T. Nguyen, K.Q. Pham, G.B. Tran

**Affiliations:** 1 Respiratory Department, Instituto Ramón y Cajal de Investigación Sanitaria, Hospital Ramón y Cajal, Madrid, Spain; 2 Medicine Department, Universidad de Alcalá, Madrid, Spain; 3 Centro de Investigación Biomédica en Red Enfermedades Respiratorias, Madrid, Spain; 4 Division of Pulmonary and Critical Care Medicine, Washington University School of Medicine, St. Louis, Missouri, USA; 5 Internal Medicine Department, Hospital General Universitario Gregorio Marañón, Madrid, Spain; 6 Department of Internal Medicine, Clínica Universidad de Navarra, Madrid, Spain; 7 Interdisciplinar Teragnosis and Radiosomics Research Group, Universidad de Navarra, Madrid, Spain; 8 Center for Hemorrhagic and Thrombotic Diseases, General and University Hospital S. Maria della Misericordia, Udine, Italy; 9 Department of Haematology, Hospital Clínico Universitario Lozano Blesa, Zaragoza, Spain; 10 Division of Cardiovascular Medicine, Brigham and Women’s Hospital, Harvard Medical School, Boston, Massachusetts, USA; 11 Thrombosis Research Group, Brigham and Women’s Hospital, Harvard Medical School, Boston, Massachusetts, USA; 12 Yale New Haven Hospital/Yale Center for Outcomes Research and Evaluation, New Haven, Connecticut, USA; 13 Faculty of Health Sciences, Universidad Católica San Antonio de Murcia, Murcia, Spain

## 1 Introduction

Pleural effusion commonly occurs in patients with acute pulmonary embolism (PE) [1]. Several studies and meta-analyses have found inconsistent results regarding the association between pleural effusion and short-term mortality after PE [[2], [3], [4]]. Recently, Zuin et al. [5] performed a meta-analysis of 4 cohort studies (2186 patients) that assessed the prognostic role of pleural effusion in patients diagnosed with acute PE. Overall, patients with pleural effusion had a higher mortality risk than those without pleural effusion (hazard ratio, 2.42). However, some of the included studies enrolled patients who were of unclear severity, and the meta-analysis of aggregate data did not allow for adjustment for confounding variables or for evaluation of the prognostic significance of pleural effusion in specific subgroups (eg, European Society of Cardiology [ESC] risk classes). Thus, the impact of pleural effusion on the short-term mortality of patients with acute PE remains unclear and requires further investigation. Registro Informatizado de la Enfermedad TromboEmbolica (RIETE) is a large international registry of patients with PE without restrictive clinical eligibility criteria. Therefore, we analyzed RIETE Registry data to evaluate the association between the presence of pleural effusion identified on chest radiograph and 30-day mortality.

## 2 Methods

This retrospective cohort study used data from the international RIETE Registry for patients enrolled from January 1, 2001, through April 29, 2025. The RIETE Registry prospectively collects information on patients with confirmed acute venous thromboembolism. Participants provided written or oral informed consent for participation in the registry in accordance with local ethics committee requirements. A previous publication has described the design and conduct of the RIETE Registry [6]. In summary, investigators at each participating site enrolled eligible patients diagnosed with acute venous thromboembolism. In RIETE, all patients were followed up for at least 3 months.

Study eligibility criteria required hemodynamic stability at presentation (ie, systolic blood pressure ≥ 90 mm Hg), and chest radiograph that allowed for assessment of pleural effusion. We used the local reading of the chest radiograph by attending physicians to determine the absence or presence of pleural effusion. Coprimary outcomes consisted of 30-day all-cause and PE-specific mortality. The RIETE Registry collects date and cause of death after PE. For patients who died, medical record review, proxy interviews when necessary, and review of autopsy reports if available assisted with determination of the date and cause of death. Local investigators adjudicated death due to PE (fatal PE) if it was (1) confirmed by autopsy or (2) clinically suspected soon after the initial PE or shortly after an objectively confirmed recurrent event, in the absence of any alternative diagnosis.

We used Kaplan–Meier survival analyses to examine the difference in time to all-cause and time to PE-related mortality between patients with and those without pleural effusion, and we assessed the difference between each group with the log-rank test. We also used logistic regression to adjust for potential confounders that included demographic variables, preexisting chronic conditions, vital signs, and right ventricular (RV) dysfunction. We also preplanned an evaluation of the prognostic significance of pleural effusion for each of the ESC risk classes (ie, low-, intermediate-low, and intermediate-high risk) [7]. Analyses were repeated in predefined subgroups of sex, age, pulmonary infarction, RV dysfunction, active cancer, and congestive heart failure.

## 3 Results

We identified 65,747 patients with objectively confirmed symptomatic PE enrolled in RIETE during the study period. Of these, 3.2% (2093/65,747 patients) were excluded because they had hemodynamically unstable acute PE. The study excluded 22,392 patients because baseline chest radiographs were unavailable. When comparing patients with (included) and those without (excluded) a chest radiograph, we did not find a statistically significant difference (ie, standardized difference < 10% in absolute value difference) between included and excluded patients regarding demographics, medical history, and clinical presentation variable data. The final study cohort consisted of 41,262 normotensive patients with confirmed PE and an available chest radiograph around the time of PE diagnosis. Eighteen percent of patients had pleural effusion on the chest radiograph at the time of PE diagnosis. Baseline characteristics of study participants with and without pleural effusion are shown in Table.

**Table:** Demographics, medical history, and presentation, stratified by the presence or absence of pleural effusion.

Clinical characteristics	With pleural effusion (*n* = 7213) *n* (%)	Without pleural effusion (*n* = 34,049) *n* (%)
Age (y), mean ± SD	67.2 ± 17.4	67.7 ± 16.5
Age >80 y	1842 (25.5)	8088 (23.8)
Female sex	3579 (49.6)	17,910 (52.6)
**Risk factors for VTE**		
Cancer^a^	1828 (25.3)	6596 (19.4)
Recent surgery^b^	990 (13.7)	3226 (9.5)
Immobilization^c^	1831 (25.4)	8072 (23.7)
**Comorbid diseases**		
COPD	979 (13.6)	4855 (14.3)
Congestive heart failure	930 (13.0)	2465 (7.3)
Recent bleeding^b^	198 (2.7)	695 (2.0)
**Clinical symptoms and signs at presentation**		
Syncope	579 (8.1)	5046 (15.0)
Chest pain	4198 (58.7)	14,974 (44.3)
Dyspnea	5696 (79.3)	27,832 (82.0)
Heart rate ≥ 110/min	1438 (20.2)	6428 (19.2)
SBP < 100 mm Hg	305 (4.2)	1528 (4.5)
**Chest radiograph findings**		
Infarction	1111 (15.4)	1670 (4.9)
Infiltrates	2063 (28.6)	5163 (15.2)
Enlarged heart	1901 (26.4)	6093 (17.9)
**CT findings**		
Central thrombus location	187 (5.7)	1735 (10.5)
RV enlargement,^d^*N* = 4793	300/720 (41.7)	2014/4073 (49.4)
**Echocardiography and cardiac biomarkers**		
RV dysfunction,^e^*N* = 5944	584/842 (69.4)	3656/5102 (71.7)
Positive troponin, *N* = 22,004	1164/3416 (34.1)	7581/18,588 (40.8)
**Laboratory tests**		
Abnormal creatinine levels (> 2 mg/dL)	1402 (19.8)	6518 (19.4)
Hemoglobin (g/dL), mean ± SD	12.8 ± 2.6	13.2 ± 2.1
**Advanced treatments**		
Systemic thrombolysis	82 (1.1)	772 (2.3)
Catheter-based therapies	47 (0.7)	143 (0.4)
IVC filter insertion	196 (2.7)	858 (2.5)

COPD, chronic obstructive pulmonary disease; CT, computed tomography; IVC, inferior vena cava; RV, right ventricle; SBP, systolic blood pressure; VTE, venous thromboembolism.

a Active or under treatment in the last year.

b In the previous month.

c Immobilized patients are defined in this analysis as nonsurgical patients who had been immobilized (ie, total bed rest with bathroom privileges) for ≥ 4 days in the month prior to pulmonary embolism diagnosis.

d RV/left ventricle chamber diameter > 1.

e RV hypokinesis and/or RV/left ventricle chamber diameter > 1.

The study had complete survival information for all (100%) patients during the 30-day follow-up period; 1972 (4.8%) of the 41,262 died within 30 days of follow-up. Five hundred forty-four patients (1.3% of the study cohort) died from PE, and 1428 (3.5% of the study cohort) died from other causes. Among the group of patients with pleural effusion, 542 (7.5%) died, whereas 1430 deaths (4.2%) occurred in the group of patients without pleural effusion (log-rank *P* < .001; Figure). Thirty-day PE-related mortality rates were 1.8% and 1.2% in those with and without pleural effusion, respectively (log-rank *P* < .001; Figure).

**Figure:**
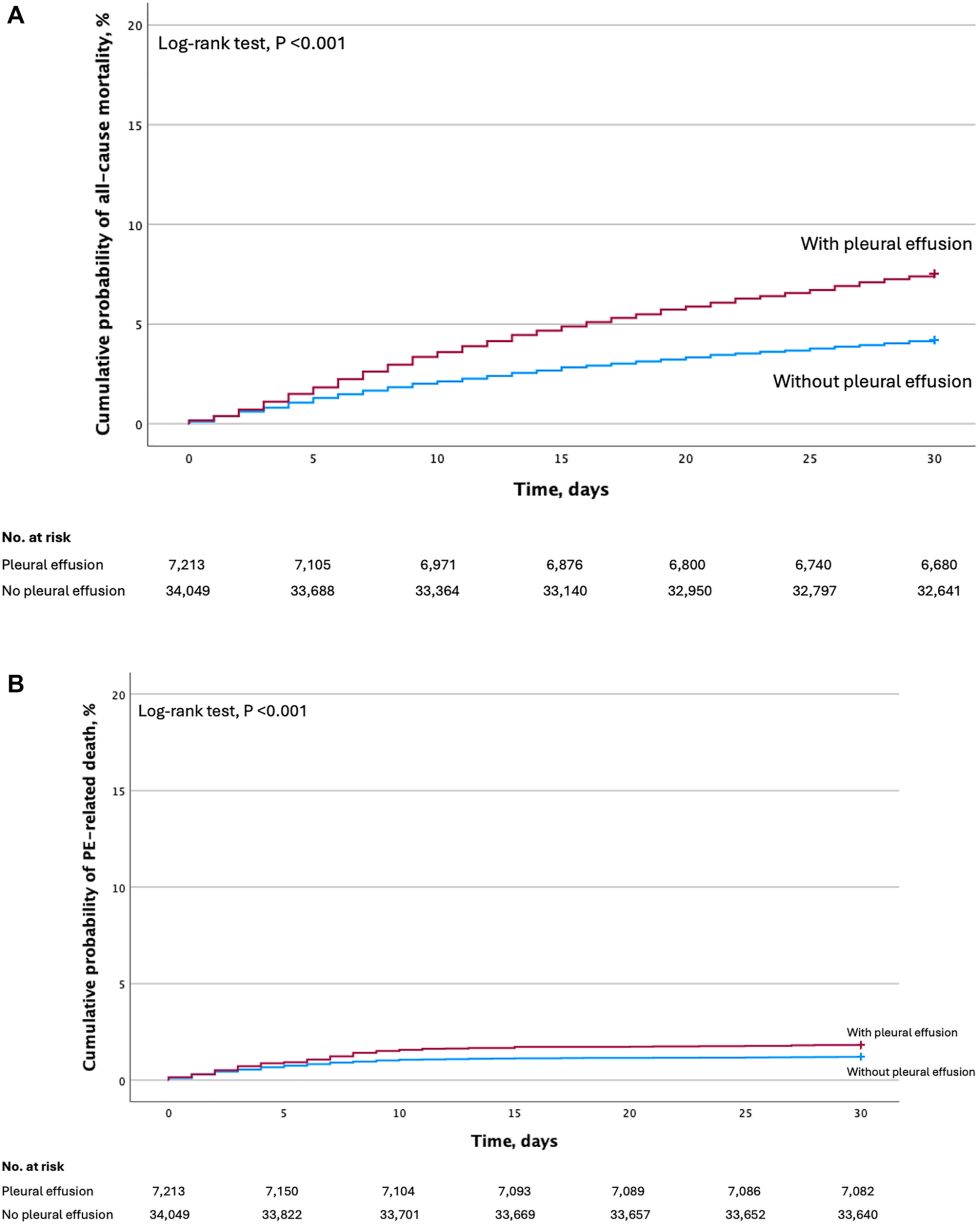
Unadjusted Kaplan–Meier 30-day mortality according to the presence or absence of pleural effusion. (A) All-cause mortality and (B) pulmonary embolism-related mortality.

After adjustment for age, sex, coexisting conditions (ie, cancer, immobilization, and chronic heart disease), vital signs (ie, systolic blood pressure and heart rate), and RV dysfunction, patients with pleural effusion had significantly higher odds of 30-day all-cause mortality than those without pleural effusion (adjusted odds ratio [OR], 1.84; 95% CI, 1.30-2.60). After adjustment, patients with pleural effusion did not have significantly higher odds of 30-day PE-related death than those without pleural effusion (OR, 0.95; 95% CI, 0.47-1.91).

The 3 ESC risk groups had a similar prevalence (16.8%-18.0%) of pleural effusion around the time of PE diagnosis. For patients with intermediate-high risk (13.0% vs 9.3%) and intermediate–low-risk (10.2% vs 5.6%) PE, but not those with low-risk PE (0.9% vs 0.8%), the presence of pleural effusion was associated with an increased unadjusted 30-day all-cause mortality rate.

Subgroup analyses according to RV dysfunction (OR, 1.73; 95% CI, 1.25-2.40), cancer (OR, 1.75; 95% CI, 1.50-2.04), and congestive heart failure (OR, 1.48; 95% CI, 1.16-1.89) had findings similar to the main analyses. However, in the subgroup of patients with pulmonary infarction (OR, 0.92; 95% CI, 0.58-1.45), the presence of pleural effusion was not associated with 30-day all-cause mortality.

## 4 Discussion

We found that pleural effusion on the chest radiograph occurred in approximately one-fifth of this nonhypotensive acute PE cohort.

The study’s most relevant prognostic finding was the near-doubling of the probability of short-term all-cause mortality associated with the presence of pleural effusion. However, pleural effusion was not associated with PE-related mortality. Given this discrepancy, we hypothesize that pleural effusion serves as a marker of overall disease severity associated with comorbidities. The finding that patients with pleural effusion had a less severe presentation of PE further supports this hypothesis. We also did not detect increased mortality associated with the presence of pleural effusion in the subgroup of patients with concomitant pulmonary infarction. Previous research has suggested that an inflammatory response more frequently causes pleural effusion associated with pulmonary infarction than central hemodynamic compromise (eg, RV dysfunction), and the latter is a major driver of mortality in more severe, proximal PE [8].

Our study might have PE treatment implications. PE patients deemed as low-risk for early adverse outcomes on the basis of a nonelevated simplified pulmonary embolism severity index score and the absence of computed tomography (CT)-assessed RV enlargement might safely undergo outpatient PE therapy [9], regardless of the presence or absence of pleural effusion. Among patients with intermediate-low and intermediate-high PE, lack of association of pleural effusion with PE-related mortality precludes the use of pleural effusion to drive the consideration of escalated PE-specific treatment, such as catheter-directed therapy.

This study contains some methodological limitations. First, the presence or absence of pleural effusion was determined based on a locally analyzed chest radiograph (ie, not chest CT), so there is a chance of diagnostic misclassification. Existing data elements do not allow us to assess the prognostic role of CT-based effusions. Second, since the RIETE Registry does not require information on the side (ie, right, left, or bilateral) and amount of pleural effusion to be systematically collected, our study could not show any subgroup analysis for such association. Third, the use of chest radiographs to identify the presence of pleural effusion and the exclusion of patients with more severe (ie, high risk) PE might explain the relatively low percentage found in our study [[10], [11], [12], [13]]. Fourth, despite a large cohort size and adjustment for key covariates, residual confounding is possible, and causality cannot be inferred.

In conclusion, among nonhypotensive patients diagnosed with acute PE, the presence of pleural effusion was an independent predictor of short-term all-cause mortality. However, an increased mortality risk was not present in the subgroup of patients with concomitant pulmonary infarction or among those with ESC low-risk PE. Further investigation is required to confirm these findings, assess possible underlying mechanisms, and determine the role of pleural effusion in the approach to PE treatment.

## Appendices

**RIETE Registry**

**Principal Investigator:** Manuel Monreal.

**RIETE Steering Committee members:** Paolo Prandoni, Benjamin Brenner, and Dominique Farge-Bancel.

**RIETE National coordinators:** Raquel Barba (Spain), Peter Verhamme (Belgium), Hugo Hyung Bok Yoo (Brazil), Radovan Malý (Czech Republic), Laurent Bertoletti (France), Sebastian Schellong (Germany), Inna Tzoran (Israel), Pierpaolo Di Micco (Italy), Abilio Reis (Portugal), Marijan Bosevski (R. North Macedonia), Lucia Mazzolai (Switzerland), Joseph A. Caprini (USA), My Hanh Bui (Vietnam).

**RIETE Registry Coordinating Center:** S&H Medical Science Service.

**Members of the RIETE Group**

**Spain:** Abad-Fernández A., Adarraga M.D., Aibar J., Alda A., Alfonso J., Álvarez-Albarrán J., Amado C., Angelina-García M., Arcelus J.I., Ballaz A., Barba R., Barbagelata C., Barrón M., Barrón-Andrés B., Bascuñana-Girón J., Beddar-Chaib F., Bencosme de Méndez E.I., Blanco-Molina Á., Chamorro N., Chasco L., Claver G., Creu-Paris J., Criado J., De Juana-Izquierdo C., Del Molino F., Del Toro J., Demelo-Rodríguez P., Díaz-Pedroche M.C., Díaz-Peromingo J.A., Dubois-Silva Á., Durán D., Escribano J.C., Falgá C., Fernández-Bermejo L.A., Fernández-Martínez de Septién C., Fernández-Reyes J.L., Ferreiro-Celeiro J., Gabara C., Galeano-Valle F., García-Ortega A., Gavín-Sebastián O., Gil-Díaz A., Gómez-Cuervo C., Gorostidi-Pérez J., Guirado L., Gutiérrez-Guisado J., Hernández-Blasco L., Hernández-Borge J., Jiménez D., Joya M.D., Llamas P., López-Brull H., López-Jiménez L., López-Miguel P., López-Núñez J.J., López-Ruiz A., López-Sáez J.B., Lorenzo A., Madridano O., Maestre A., Marchena P.J., Menéndez-Sánchez C., Mercado M.I., Moisés-Lafuente J., Monreal M., Monzón-Escribano L., Moreno-Fernández A., Navas M.S., Nieto J.A., Núñez-Fernández M.J., Olid M., Ordieres-Ortega L., Ortiz M., Osorio J., Osoro-Suárez C., Otálora S., Pagán J., Parra-Caballero P., Pedrajas J.M., Pérez-Pinar M., Pérez-Ductor C., Peris M.L., Pesce M.L., Porras J.A., Prieto-Gañán L.M., Puche G., Revilla D., Rivas A., Rivera-Cívico F., Rivera-Gallego A., Roca-Toledo M., Rodríguez-Cobo A., Ruiz-Artacho P., Ruiz-Giménez N., Sánchez-Camacho M., Sancho T., Sierra-Palomares G., Sigüenza P., Sindín-Martín L., Solé A., Suárez-Fernández S., Tascón-Rodríguez R., Terés-Pueyo L., Tirado R., Toda M.R., Tolosa C., Torregrosa-Ruíz P., Trujillo-Santos J., Tudela L., Valle R., Varona J.F., Vega-Romero E., Vidal G., Villares P., and You J.S.; **Austria:** Ay C., Nopp S., and Pabinger I.; **Belgium:** Erard M., Van Thillo Q., and Verhamme P.; **Brazil:** Rocha A.T. and Yoo H.H.B.; **Colombia:** Carbonell S.E., Gómez-Mesa J.E., Montenegro A.C., and Roa J.; **Czech Republic:** Grenar P., Hirmerova J., Malý R., and Válková T.; **France:** Accassat S., Bertoletti L., Bura-Riviere A., Catella J., Chopard R., Couturaud F., Crichi B., Espitia O., Le Mao R., Leclercq B., Mahé I., Millet G., Moustafa F., Plaisance L., Poenou G., Sarlon-Bartoli G., and Versini E.; **Germany:** Schellong S.; **Iran:** Jenab Y., Khodayari A., Sadeghipour P., and Yadangi S., **Israel:** Brenner B. and Tzoran I.; **Italy:** Barillari G., Basaglia M., Bilora F., Bissacco D., Bortoluzzi C., Bortoluzzi M., Brandolin B., Buso G., Casana R., Ciammaichella M.M., Di Micco P., Guida A., Imbalzano E., Lambertenghi-Deliliers D., Licci A., Marcon C., Mastroiacovo D., Mazzarelli U., Mumoli N., Pesavento R., Pizzuti V., Poz A., Prandoni P., Scarinzi P., Siniscalchi C., Visonà A., Vo Hong N., and Zalunardo B.; **Latvia:** Cesnieks H., Kigitovica D., and Skride A.; **Mexico:** Flores-Ramírez C., Mendoza Romo-Ramírez M.Á., and Tarín-Recéndez D.; **Portugal:** Fonseca S., Martins M., Pereira-Fontes C., and Soeiro B.; **Republic of North Macedonia:** Bosevski M. and Zdraveska M.; **Switzerland:** Barco S. and Mazzolai L.; **USA:** Angiolillo D.J., Bikdeli B., Caprini J.A., Khalil A., Ortega-Paz L., Parmar G.M., and Tafur A.J.; **VIetnam:** Bui M.H., Nguyen S.T., Pham K.Q., and Tran G.B.

## References

[1] Findik S. (2012). Pleural effusion in pulmonary embolism. Curr Opin Pulm Med.

[2] Choi S.H., Cha S.I., Shin K.M., Lim J.K., Yoo S.S., Lee S.Y. (2017). Clinical relevance of pleural effusion in patients with pulmonary embolism. Respiration.

[3] Rodríguez-Núñez N., Gude F., Ferreiro L., Landín-Rey E., Carreiras-Cuiña M., Otero B. (2024). Pleural effusion in acute pulmonary embolism: characteristics and relevance. BMJ Open Respir Res.

[4] Levy O., Fux D., Bartsikhovsky T., Vosko S., Tishler M., Copel L. (2020). Clinical relevance of bilateral pleural effusion in patients with acute pulmonary embolism. Intern Med J.

[5] Zuin M., Rigatelli G., Turchetta S., Pasquetto G., Roncon L., Bilato C. (2022). Mortality risk in patients with pulmonary embolism with pleural effusion. Am J Cardiol.

[6] Bikdeli B., Jimenez D., Hawkins M., Ortíz S., Prandoni P., Brenner B. (2018). Rationale, design and methodology of the computerized registry of patients with venous thromboembolism (RIETE). Thromb Haemost.

[7] Konstantinides S.V., Meyer G., Becattini C., Bueno H., Geersing G.J., Harjola V.P. (2020). 2019 ESC guidelines for the diagnosis and management of acute pulmonary embolism developed in collaboration with the European Respiratory Society (ERS). Eur Heart J.

[8] Stein P.D., Henry J.W. (1997). Clinical characteristics of patients with acute pulmonary embolism stratified according to their presenting syndromes. Chest.

[9] Mathis G., Blank W., Reissig A., Lechleitner P., Reuss J., Schuler A. (2005). Thoracic ultrasound for diagnosing pulmonary embolism: a prospective multicenter study of 352 patients. Chest.

[10] Elliott C.G., Goldhaber S.Z., Visani L., DeRosa M. (2000). Chest radiographs in acute pulmonary embolism. Results from the International Cooperative Pulmonary Embolism Registry. Chest.

[11] Bynum L.J., Wilson J.E. (1978). Radiographic features of pleural effusions in pulmonary embolism. Am Rev Respir Dis.

[12] Karabulut N., Kiroğlu Y. (2008). Relationship of parenchymal and pleural abnormalities with acute pulmonary embolism: CT findings in patients with and without embolism. Diagn Interv Radiol.

[13] Jiménez D., Bikdeli B., Rodríguez C., Muriel A., Ballaz A., Soler S. (2023). Identification of low-risk patients with acute symptomatic pulmonary embolism. Arch Bronconeumol.

